# X-Rays and Blood

**Published:** 1906-05-05

**Authors:** 


### X-RAYS AND BLOOD.

**The Action of x-rays upon Blood and Hæmopoietic Structures.**—While the effects of the *x*-rays upon the skin can be appreciated and watched, it is only by careful and frequent observation that the effects upon the internal tissues can be assessed. It is of the greatest value that we should know what actually occurs in the blood when the *x*-rays reach it, for it is only by an absolute knowledge of the action and reactions attending this form of treatment in leukæmia and allied disorders, that it will be possible to place the *x*-rays in their proper position in therapeutics. Numerous observers have been working at the effects produced in animals in a healthy state. The observations made are not markedly at variance, but the deductions made are widely different. The rays cause a marked diminution in the number of leucocytes in the peripheral circulation, though immediately after the raying there may be a temporary increase, brought about by a greater number of polynuclear cells finding their way into the blood-stream. The lymphocytes seem to be the most susceptible to the action of the rays, being affected first and most intensely. There does not seem to have been any systematic work carried out as yet to ascertain if the cells are stored anywhere, although Ledingham has observed that the various organs, in a case of leukæmia, were rich in somewhat differing kinds of cells. The liver presented the interacinar capillaries almost choked with leucocytes of the large basophile type, while the marrow of the sternum and ribs presented small and large lymphocytes in the greatest numbers. There is undoubted evidence that destruction takes place of the white cells, though the red cells are not altered in colour or in shape. The destruction is rather inferred from the diminution of the cells than actually seen, though, in animals, degeneration phenomena have been observed in the white cells, with fragmentation of the nucleus in the polynuclear cell, and degeneration of the protoplasm, as shown by the poor staining reaction, the indistinct outline and the occasional disintegration of the cells and absolute refusal to take the stain. Gramegna and Quadrone have found that the coagulability and specific gravity of the blood are increased while the hæmoglobin suffers no change, and on this account they are led to recommend the use of the rays in hæmophilia and allied hæmorrhagic disorders. There is also an increase in the excretion of uric acid and phosphoric acid in some cases, though apparently not in all. On the hæmopoietic organs there is also an appreciable change. The lymphatic glands, the spleen, and the marrow of bones all show characteristic alterations. Arneth goes so far as to hold that he has proved that in myeologenous leukæmia there is not so much a destruction of the white cells by the *x*-rays as a lessened demand for their production. He considers that in leukæmia itself there is not only an immense leucocyte destruction, and a correspondingly great demand for their production, but that there is a special stimulus to the hyperplastic development of the blood-forming organs, perhaps due to some as yet hypothetical virus. As bearing upon this subject, a case recorded at length by Ledingham is of great interest. His patient was rayed for leukæmia, and the leucocytes fell from 200,000 to 20,000 per cmm. in six weeks. After the suspension of the rays they returned gradually and irregularly, only reaching one-third of the original amount in three months. From this he makes the inference that the rays had exerted an inhibitory influence on the proliferative functions of the blood-forming organs. On resuming the *x*-rays a fresh fall occurred in the leucocyte count until in six weeks they were subnormal (7,000), a rise subsequently occurring to 12,000. At this period of leukopenia the patient contracted influenza. The temperature rose to 101° F., bronchitis ensued and a further rise occurred to 102°. Death occurred suddenly fourteen days after the commencement of the intercurrent affection. Now, as a rule, in leukæmia, intercurrent affections produce a great fall in the number of leucocytes, in this case they increased to 43,000, a rather vigorous reaction, but one in the direction usual to otherwise healthy persons. Heineke has shown that in mice which have been exposed to the rays there is a destruction of lymphoid follicles in the spleen and glands; the nuclear detritus is taken up by the phagocytes, and the nodes replaced by connective tissue and epithelioid cells. Prolonged exposure brings about a destruction of the granular cells in the bone marrow. Mosse and Milchner have shown that bone marrow is profoundly influenced, the cells of the neutrophile series being greatly reduced, while those left have shown a great deficiency in granules. Halber and Linser, after prolonged exposure of small animals, have depleted the circulation entirely of leucocytes, though in larger animals they found it difficult to reach a figure below 1,500 to 2,000 per cmm. Fatal results have ensued which they have attributed to the leucotoxins liberated from the broken-down leucocytes. The serum of *x*-rayed animals has been shown to have the power of destroying leucocytes in the blood of fresh animals. It would seem that the lymphocytes are the most easily influenced by the rays. Ledingham asks: "Is the diminution of the leucocytes in the circulating blood attributable to increased destruction of the cells in the hæmopoietic organs, or to decreased output?" If it is due to increased destruction, then there should be an increase in the uric acid excretion. In his case the uric acid, the total nitrogen, and the total phosphoric acid were estimated, but no relation could be established between this extra excretion and the decrease in the leucocytes. The total phosphoric acid bore the usual proportion to the total nitrogen. This, in his opinion, militates against the view that there is an increase in the destruction of white corpuscles. The bulk of the chemical evidence lies in favour of the view that the *x*-rays exert an inhibitory influence on the hæmopoietic organs. These organs Ledingham found in his case to be profoundly altered, but the most remarkable change was the substitution of proliferating undifferentiated basophile myelocytes for the fully formed neutrophiles usually found in the spleen of chronic splenomedullary leukæmia. Attention is drawn to the following facts: During the first raying the percentage of neutrophiles increased relatively, that of the lymphocytes decreased. On discontinuing, the lymphocytes increased again. On renewing the rays the neutrophile percentage increased, while the lymphocyte percentage decreased temporarily. When the leucocyte count approached the normal the polynuclear percentage fell markedly, while that of the lymphocytes rose. So that at first, with a high count, the *x*-ray effect is mainly on the lymphocytes, the spleen and lymph glands functionating most actively; hence, in spite of an actual decrease in the neutrophiles, the percentage was raised. At the leucopenic stage, however, the neutrophiles were produced in less and less amount. It is at this stage when it may prove dangerous to continue with the rays lest a dangerous leucotoxic effect ensue. There seems to be no satisfactory explanation of the enormous clinical improvement in the general condition of the patients under the rays; it is difficult to bring it into relationship with the blood changes without falling back upon the hypothetical parasite of Senn and Ahrens. Some information may be obtainable from a study in the opsonic index of patients. At present Ledingham has found that the opsonin in leukæmic serum (splenomedullary) is normal in amount, but myelocytes have an exceedingly feeble phagocytic power, as tested by staphylococci and tubercle. Any increase, therefore, in the number of ripe polynuclears in the circulating blood must also render the normal supply of opsonin more available for purposes of efficient phagocytosis and removal of effete matter from the tissues. In chronic lymphatic leukæmia the reverse seems to hold. The polynuclears, in two cases examined, had double the phagocytic power of a healthy individual, and the true opsonic index as measured by the patient's own serum and phagocytes was relatively high.

